# Supplementary material for: Acceptance and compliance with micronutrient powder (MNP) among children aged 6–23 months in northern Nigeria
Source: PLOS Glob Public Health. 2022 Oct 17;2(10):e0000961. doi: 10.1371/journal.pgph.0000961 (PMC10022258; doi:10.1371/journal.pgph.0000961)
Supplement: S1 Dataset — (ZIP) [file pgph.0000961.s008.zip › S8_Dataset/Direct Observations.zip!/Direct Observations/040715_K_BK_AD_2_DO.pdf]

1.1.1 Instrument F –Direct Observation Form

<<Instrument F Direct Observation Form>>
**PI Name:** PragyaMathema,Stephen Kodish<br>**Study Title:** Formative research to develop an infant and young child feeding (IYCF) intervention with micronutrient powder (MNP) for children aged 6 – 23 months<br>**PI Version/Date:** Version1.0/January 24, 2015

Date: 04/07/15Location: Bakan Yadiya

Data Collector: Amina Duniya

Main goals of this direct observation are to answer the following questions:
1. In <u>this</u> household, what would be the best way(s) to give nutritional supplements to <u>young children</u>? Why?
2. In <u>this</u> household, describe the food sharing that you observe throughout the meal. Are there any <u>predictable</u> patterns?
3. In <u>this</u> household, who is favored and how is this favoritism expressed during meal preparation and eating?

Household Composition Table (Fill out <u>as</u> you do data collection)

INITIALS	SEX (M/F)	AGE (estimated)	Relationship to household	Description (not necessary)
BM	F	20years	Mother	Wearing a white and red strip blouse, yellow wrapper and yellow head tie
FH	M	40years	Father of household	Slim, dark in complexion and wearing a blue kaftan
BB	M	9months	Baby boy	Wearing a blue top only. Has a scar on the forehead and nose
FW	F	30years	First wife of husband	Wearing a yellow blouse and multi colored wrapper and head tie
SI	F	17years	Sister inlaw	Tying a yellow wrapper on her chest with a black singlet
FS	M	2years	First son of mother	Wearing a blue top no pants

SS	F	3years	Step daughter to BB(father's daughter)	
GF	M	50years	Grandfather	Has Grey hair, wearing a pink and cream colored kaftan
FD	F	4years	First daughter of mother	Black skirt and purple blouse. Dark in complexion

PARTICIPANT ID: _________

TIME	ACTOR INITIALS	RECIPIENT INITIALS	WHAT HAPPENED	FOOD	Reactivity
10:00	FH	BB	Father playing with BB inside the compound		
	BM		Pounding millet with FW		
10:05	FM	BM	Gives BB to FW and goes out		
	FW	BB	Plays with BB and BM still pounding		
10:15	FW	BM	Baby crying and FW gives him back to BM		
	BM	BB	BM collects BB and breast feeds him	Breast milk	
	BM	BB	Falls asleep and BM puts him on her back		
10:20	BM		Continues pounding millet Finished pounding millet into powder form and pours into a calabash		
	BM		Pours water into millet flour and sieves		
	FH		Comes back and sits in then compound		
	FH		Playing with older son		
10:25	BM		Pours millet flour into mortar and uses then pestle to make a hard dough		

TIME	ACTOR INITIALS	RECIPIENT INITIALS	WHAT HAPPENED	FOOD	Reactivity
10:30	BM		Fetches dough and goes into the room while baby still sleeps on her back		
10:35	BM		Doing house chores while the baby is sleeping on her back		
10:40	FW		FW visits BM in her room while BB is still sleeping on BM's back		
10:45	FW	BM	Still with BM		
10:50	BM		BM goes outside to wash dirty pot		
10:55	BB		Still sleeping on BM's back		
11:00	BM	FW	Still discussing with FW		
11:05	BM	FW	Still discussing with FW		
11:10	FW		Leaves to her room		
11:15	BB		Wakes up from sleep		
11:20	BM	BB	Plays with BB		
11:25	SI		Comes into BM's room		
11:30	FW		Comes back into BM's room		
11:35	BB		Still playing with BM		
11:40	FW		Spits saliva on the floor		
11:45	BM		Discussing with SI and FW		
11:50	BB		Playing on his own with a chair		
11:55	FW		Leaves to go to her daughter's house		
12:00	BM		SI leaves and BM continues playing with BB		

TIME	ACTOR INITIALS	RECIPIENT INITIALS	WHAT HAPPENED	FOOD	Reactivity
12:05	BB		BB starts crying		
	BB		Refuses to take breast milk from BM		
	BB		Continues to play and crawl around the room		
	BM		Goes out to visit SI		
12:10	BB		Crawls to FH and begins to play		
	FH		Playing with BB in the compound		
12:15	BB		Still playing with FH		
12:20	BB		Still playing with FH		
	BM	BB	Comes back and carries BB to her room		
12:25	BM	BB	Playing with BB in her room		
12:30	BM	BB	Gives him breast milk to drink<br>Finished drinking breast milk and continues playing		
12:35	BB		Still playing		
12:40	BB		Playing with BM		
	FS	BB	Comes in and begins to play with BB		
12:45	FS	BB	Still playing together		
12:50	BM	BB	Carries BB to FH		
	FH	BB	Playing with BB		
12:55	BB		Still playing together		
1:00	BB		Playing with FH		
1:05	BB		Playing with FS		

TIME	ACTOR INITIALS	RECIPIENT INITIALS	WHAT HAPPENED	FOOD	Reactivity
1:10	BM		Comes back and begins to wash plates		
1:15	BM	BB	Finished washing plates and begins to play with BB		
1:20	BM	BB	Gives him kunu to drink	Kunun gero	
1:25	BB		Finished drinking kunu and begins playing with FS		
1:30	SS	BB	Carries BB to play with under the tree		
1:35	BB		Playing alone under the tree		
	BM		Pounding guinea corn to remove the back		
1:40	BB		Still playing by himself		
	BM		Fetches pounded guinea corn from the mortar and washes it		
1:45	BB		Plays with water used in washing guinea corn		
	BM		Fetches clean water and washes BB's body		
1:50	BM	BB	Breast feeds BB	Breast milk	
1:55	BB		Finished feeding and playing on BM's lap		
2:00	BB		Still playing		
2:05	BM	BB	Keeps BB under the tree to play		
	BM	FS	Baths FS		
2:10	BB		Still playing by himself under the tree		
	BB		Starts crying and crawls to BM's room		
2:15	BB		Playing by himself in BM's room		
2:20	SS	BB	Comes back and begins to play with BB		
2:25	BM	BB	Picks BB from the floor and begins		

TIME	ACTOR INITIALS	RECIPIENT INITIALS	WHAT HAPPENED	FOOD	Reactivity
			to play with BB		
2:30	BM	BB	Feeds BB with breast milk	Breast milk	
	BB		Finished feeding and continues to play		
2:35	BM	BB	Goes back to sucking the mother's breast	Breast milk	
2:40	BB		Continues to play under the tree		
2:50	BB		Still playing		
	BM		Peeling cassava and watching BB playing		
2:55	BB		Eats cassava	Cassava	
3:00	BB		Plays with cassava while BM still peels cassava		
3:05	BB		Still playing		
	FH	FS&BB	Comes back and starts playing with BB&FS		
3:10	FH	BB	Still playing together		
	BB		Playing on his own		
	FH	BB	Watching BB play		
3:20	BB		Still playing		
3:25	FH		Gets up and goes outside the house		
3:30	BM	BB	Carries BB into the room brings him back and drops him under the tree to play		
	BM		Begins to pound guinea corn		
3:35	BB		Still playing		
	BM	BB	Gives him water to drink		
	BB		Crawls into GF's hut		

TIME	ACTOR INITIALS	RECIPIENT INITIALS	WHAT HAPPENED	FOOD	Reactivity
3:40	BM	BB	Carries him from GF's hut and keeps him with GF to play outside		
	BB		Crawls away from GF to under the tree		
	BM		Still pounding		
3:45	BB		Still playing under the tree		
	BM	BB	Picks BB up and puts him on her back and continues pounding guinea corn		
3:50	BM	BB	Drops him under the tree to continue playing		
	BM		Sieves the guinea corn flour in preparation fro dinner		
3:55	FH		Comes back and picks BB up to play with		Picks him up too often
4:00	BM		Trying to make fire for dinner		
			Puts BB on her back		
	BB		Refuses to stay on BB's back and now playing on the floor		
4:05	BB		Still playing on then floor		
4:15	BB		Still playing		
	BM		Finally makes the fire and puts a pot of water on it to boil		
	BM		Mixes the MNP product with very thick kunun gero. The kunun gero is warm	Kunun gero	
4:20	BM	BB	Breast feeding the baby because he starts to cry before she could finish mixing the food with MNP	Breast milk	
	BM	BB	Gives him kunun gero mixed with MNP<br>Due to the thickness of the kunu, BM uses her finger to fetch the kunu	Kunun gero	

			and give to BB		
4:25	BM	FD,FS&BB	BM allows FD and FS to eat same food mixed with MNP for BB	Kunun gero kixed with MNP	
4:30	BM	BB	BM finally stops FD and FS from eating the food mixed with MNP and feeds BB alone	Kunun gero kixed with MNP	
4:40	BM		Performs ablution for prayers while BB IS ON HER BACK		
	FH		Comes and carries BB to go play		
4:43	FH		Drops BB and goes out again		This shows that he is dropping him for my sake
4:45	BB		Playing on the floor		
	BM	BB	BB starts crying and BM picks him up an breast feeds him	Breast milk	
4:50	BB		Begins to sleep while sucking	Breast milk	
4:55	BB		Still sucking and sleeping		
4:58	BB		Sleeps off finally and releases BM's breast		
5:00	BM		Puts him down on the mat to sleep		
5:05	BM		Pounding guinea corn flour for tuwo		
	BB		Still sleeping		
5:10	BB		Still sleeping		
	BM		Still pounding		
5:20	BB		Still sleeping		
	BM		Waiting for water to boil		
5:30	BB		Still sleeping		

	BM		Goes to FW's house and greets her		
5:35	BM		Comes back from FW's house and is fetching water		
5:40	BB		Still sleeping		
	BM		Finish fetching water while waiting for pot of water to boil		
	BB		Wakes up from sleep		
5:45	FD	BB	Fetches some water and gives BB to drink		
	BM	BB	Breast feeds the baby	Breast milk	
5:50	BM	FW	Gives BB to FW to hold<br>Refuses to stay with FW		
5:55	BM	BB	Breast feeds the baby	Breast milk	
6:00	BB		Starts crying and BM carries him		

Primary Questions (Complete after you complete data collection):

From what you have seen observing THIS HOUSEHOLD, please answer the following questions in detail:

Question	Write your detailed response here
1. In this household, what would be the best way(s) to give nutritional supplements such as an MNP to young children to ensure compliance?<br><br>Why?	The best way to give the MNP is through the mother because she Is the one who feeds the child directly and also the senior wife (1st wife) of the husband because it seems she seems to have huge influence on the mother of the child.
2. In this household, who is favored during meal preparation or feeding?<br><br>How is this favoritism expressed?	The baby is favored the most because the mother feeds him directly during his meal time whereas the other children are given their food and told to go and eat wherever they want to.

3. How could we promote the MNP supplement for this family that would attract their attention?<br><br>Who would they listen to?	The product can be promoted through then involvement of the community leader the husbands. Every member of the community listens to the community leader and every wife listens to her husband.
4. In this household, what would be the best ways to promote the MNP but limit how much it would be shared?<br><br>Why?	The best way to promote it would be through proper enlightenment about the benefits associated with the product and what they stand to lose if not used for him/her alone
5. What other issues and concerns did you see with this family that are important for us to consider with regard to introduction of an MNP?<br><br>Why?	There were many children in the house because it is an extended family consisting of many brothers and their wives and children. Therefore, when introducing the program, there is need to emphasize to the parent not to share the product with the other children.

6. Based on your experience observing this child today, do you believe reactivity negatively impacted your ability to see the food-related behaviors of the child?<br><br>Please explain your answer in detail.	No, I don't think so because the majority of the reactivity I saw had to do with the father trying to pretend that he plays with the child often

NOTE

The mother usually gives the baby the MNP in the evening and when asked why she said she does it that way because by evening time, the child would be very hungry, therefore once she gives the kunu to him, he takes it without hesitation and finishes it all.
